# Detection and score grading for prostate adenocarcinoma using semantic segmentation

**DOI:** 10.1371/journal.pone.0331613

**Published:** 2025-09-19

**Authors:** Kasikrit Damkliang, Paramee Thongsuksai, Thakerng Wongsirichot, Kanita Kayasut

**Affiliations:** 1 Division of Computational Science, Faculty of Science, Prince of Songkla University, Hat Yai, Songkhla, Thailand; 2 Department of Pathology, Faculty of Medicine, Prince of Songkla University, Hat Yai, Songkhla, Thailand; The University of Texas, MD Anderson Cancer Center, UNITED STATES OF AMERICA

## Abstract

Prostate cancer is a major global health challenge. In this study, we present an approach for the detection and grading of prostate cancer through the semantic segmentation of adenocarcinoma tissues, specifically focusing on distinguishing between Gleason patterns 3 and 4. Our method leverages deep learning techniques to improve diagnostic accuracy and enhance patient treatment strategies. We developed a new dataset comprising 100 digitized whole-slide images of prostate needle core biopsy specimens, which are publicly available for research purposes. Our proposed model integrates dilated attention mechanisms and a residual convolutional U-Net architecture to enhance the richness of feature representations. Class imbalance is addressed using pixel expansion and class weights, and a five-fold cross-validation method ensures robust training and validation. In model ensemble evaluation, the model achieves an average Dice of 0.87 and accuracy of 0.92 on the cross-validation held-out folds. When applied to completely unseen, external test data, the model demonstrates an average Dice of 0.64 and accuracy of 0.81. Segmentation and grading results were validated by a team of expert pathologists. Based on experimental results, this study demonstrates the potential of our proposed method and model as a valuable tool for the detection and grading of prostate cancer in clinical settings.

## Introduction

In 2020, there were an estimated 18.1 million new cancer cases worldwide, including 9.3 million cases in men and 8.8 million in women. Prostate cancer (PCa) is the fourth most commonly diagnosed cancer overall in both sexes, accounting for 7.3% of all cases, following breast, lung, and colorectal cancers [1]. Among men, PCa is the second most common cancer, with an estimated 1.4 million new cases. In terms of mortality, cancer was responsible for nearly 10 million deaths worldwide, with PCa accounting for 375,304 of these fatalities [1].

The highest incidence rates of PCa are observed in well-developed Western countries such as the United States (age-standardized incidence rate (ASR): 75.2 per 100,000 person-years) [2]. The incidence rates are remarkably lower in non-Western countries, including Thailand (ASR of 12.5 per 100,000 person-years) [2]. Despite this, PCa is among the top five leading cancers in Thailand, alongside liver, lung, colorectal, and non-Hodgkin lymphoma [2]. The incidence and mortality rates of PCa have shown a declining trend in many Western countries, but have witnessed an increase in Eastern Europe and Asia [3]. In Thailand, the incidence has continued to increase, with an estimated annual percent change of 5.3% and is expected to double the 2013 rate by the year 2030 [4].

The vast majority of PCa is adenocarcinoma, which originates from the secretory epithelium of prostate glands. It often develops without noticeable symptoms in its early stages [5,6]. An increased blood level of Prostate-Specific Antigen (PSA) in individuals is indicative of the need for a prostate biopsy to confirm or exclude the presence of cancer. For pathological evaluation, if adenocarcinoma is present, the tumor is morphologically graded based on its architectural pattern, ranging from simple to complex, namely Gleason Patterns (GP) 3 to 5. The numbers of the two most dominant patterns are then summed up to determine the Gleason Score (GS), which ranges from 6 to 10, e.g., GS = 6 (3 + 3) or GS = 7 (3 + 4 or 4 + 3) [5]. The higher the GS, the more likely that the cancer will grow and spread quickly; therefore, the accurate determination of GP is important.

In this work, the primary objective was to discern between benign, GP3, and GP4 tissues in multi-class semantic segmentation. Our dataset was composed of 100 unique digitized whole-slide images (WSIs) in Hematoxylin & Eosin (H&E) staining through expansion and augmentation techniques. We adopted an ensemble approach and a statistical method to analyze model performances in order to select the best method for inferring unseen WSIs. We introduce semantic segmentation for GP3, GP4, and GS in slide-level, potentially serving as an automatic screening tool for the detection and grading of PCa in tissue biopsies.

The main contributions of this work are as follows:

- Proposed an optimal multi-class pixel-level semantic segmentation method for detecting and grading adenocarcinoma in PCa, specifically distinguishing between GP3 and GP4, using our publicly available dataset of 100 digitized WSIs.
- Developed a model combining dilated attention with a residual convolutional U-Net to expand the receptive fields of feature maps.
- Implemented techniques to address class imbalance, including pixel expansion and computed class weights, and employed a five-fold cross-validation method to ensure robust training and validation.
- Achieved high performance metrics on both validation and separate testing sets, demonstrating the model’s effectiveness, generalization, and potential as a tool for PCa detection and grading in clinical settings, with qualitative results validated by pathologists.

## Literature reviews

In PCa analysis from histological WSIs, Duran-Lopez et al. [7] introduced a novel patch-scoring algorithm for filtering irrelevant areas in PCa tissue samples. They developed a custom convolutional neural network (CNN) for binary classification, effectively identifying and visualizing malignant regions in a heatmap. The impact of stain normalization on reducing color variability between scanners was critically evaluated. The system demonstrated high accuracy (99.98%) and F1 score (99.98%), validated through a 3-fold cross-validation method.

Recently, the Vision Transformer (ViT), proposed by Dosovitskiy et al. [8], introduced the idea of transforming and analyzing images as word embeddings. It has since been adapted for various medical image segmentation tasks [9], demonstrating an exceptional ability to capture global contextual information. The adoption of Transformer models, initially renowned in natural language processing, has recently shown promising results in medical imaging, including PCa analysis [10,11]. Transformers excel in image registration tasks because their self-attention mechanism allows for accurate spatial correlation between images. This approach, first introduced by Chen et al. [12], is especially effective in aligning moving and fixed images by precisely mapping their spatial features.

Practical architectures, dominated by the CNN-based U-Net model, have been proposed in studies utilizing prostate biopsy H&E stained samples [13–15]. Li et al. [13] presented an automated Gleason grading and pattern region segmentation method for pathological images across five grades. They employed a novel architecture merging spatial pyramid pooling with a standard CNN-based multiscale network, achieving precise segmentation. Their method obtained a mean intersection over union of 77.29% and an overall pixel accuracy of 89.51%. This work underscored the efficacy of combining diverse deep CNN architectures for consistent and reproducible Gleason grading in PCa.

Marginean et al. [14] developed an AI algorithm using machine learning and CNNs for improved standardization in Gleason grading of PCa biopsies. Trained on 698 sections from 174 patients and tested on 37 sections from 21 patients, the algorithm demonstrated high accuracy in cancer detection (100% sensitivity, 68% specificity). It reliably detected Gleason patterns 3 and 4 with 89% and 91% sensitivity and 77% and 79% specificity, respectively, comparable to expert pathologists.

Ahmad et al. [15] proposed an attention-based enhanced U-Net architecture with a residual block for nuclei segmentation in 20 cancer sites, including the prostate. Three different H&E stained digital histological datasets were used to develop and validate the proposed architecture. They achieved the highest Jaccard index of 0.78, and an F1 score and recall of 0.84 across all datasets.

In this work, we focus on enhancing our CNN-based U-Net network by incorporating dilation rates (DRs) in the convolutional layers, inspired by Gudhe et al. [16]. The DRs, defining the spacing between kernel elements in the filters, allow our network to capture a wider range of information from a larger receptive field. This is achieved without increasing the number of parameters, thus avoiding additional computational intensity. We carefully configured these DRs to suit our hardware capacities, ensuring optimal performance without adding computational burden.

## Materials and methods

This section outlines the materials, proposed methods, and the architecture used in this study’s training process. All protocols strictly adhered to the Declaration of Helsinki and received approval from the Ethics Committee of the Faculty of Medicine, Prince of Songkla University, Thailand (EC. NO. 64-556-19-2). The approval included a waiver for the need for informed consent concerning the analysis of human prostate tissue. The data were accessed during the approved period, from December 17, 2021, to December 16, 2022.

### Data acquisition

In this work, histologic tissue slides from 100 patients, diagnosed with acinar adenocarcinoma of the prostate, were sourced from the Department of Pathology archives at the Faculty of Medicine, Prince of Songkla University. These tissue samples were collected from patients who underwent transrectal ultrasound and biopsy procedures between 2018 and 2021. A single slide was selected per patient, exhibiting a significant proportion of GP3 or GP4. Each slide typically contained 2 to 6 tissue cores, while those with marked tissue distortion were excluded from the dataset. The summary of data acquisition criteria was presented in [17].

The histologic slides underwent review under a microscope by two senior pathologists, following the guidelines outlined in the World Health Organization (WHO) Classification of Tumors of the Urinary System and Male Genital Organs - 2016 by Humphrey et al. [18]. Initially, the first pathologist (Paramee Thongsuksai) assessed the Gleason grades, cross-referencing them with the original diagnosis. In cases of discrepancy, the slides underwent further evaluation by the second pathologist (Kanita Kayasut), and any disparities were resolved through discussion using a multi-head microscope to reach a consensus. No reference panel was utilized during this stage.

### Data preprocessing

**Fig 1:**
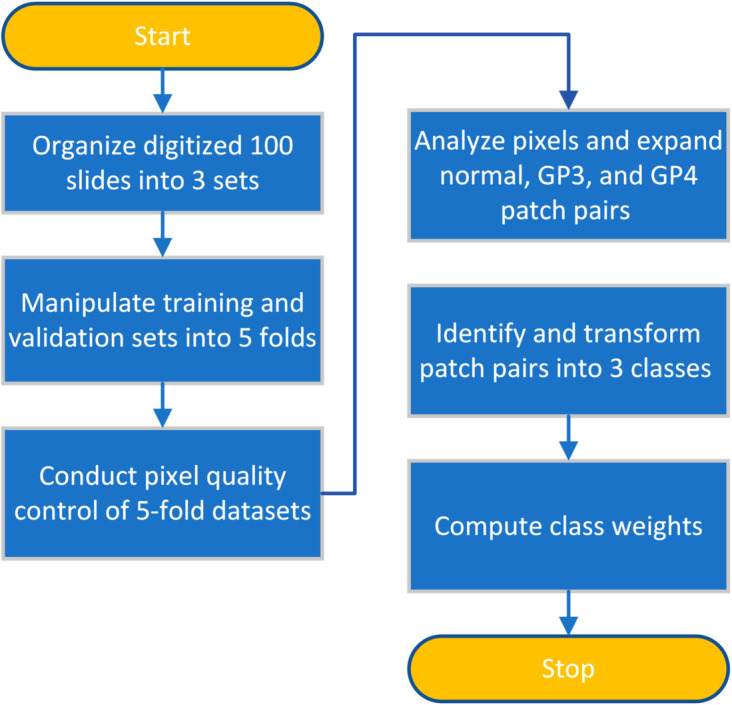
Flowchart illustrating the preprocessing steps for the PCa slide dataset including division into sets, fold manipulation, and class weight calculation.

A visual representation of the dataset preprocessing steps is provided in Fig 1. The dataset comprised 100 unique slides obtained from individual patients, carefully selected, annotated, and organized. For testing purposes, 20 slides were randomly chosen to ensure their exclusion from model exposure. The remaining 80 slides were split into training and validation sets, maintaining an 80:20 ratio.

Selected histologic slides were digitized at 40X objective power (0.25 μm/pixel) using a Leica Aperio AT2 280 scanner, yielding pyramidal WSIs with four down-sampling levels and an 8-bit depth [17]. Adhering to standard pathology practices, we extracted 256 x 256 pixel image patches at 20X magnification (level 4 down-sampling) for analysis, creating corresponding ground truth masks for the multi-class semantic segmentation in this work.

Five folds were created from these slides, and image patches with a pixel ratio of less than 20% were excluded to maintain pixel quality control. Each fold comprises 64 slides for training, 16 for validation, and 20 for testing, ensuring the uniqueness of the validation set within each fold.

In this study, our discrimination methods targeted GP3 and GP4 tissues. As part of the preprocessing steps, patches containing GP5 pixels were eliminated.

To address class imbalance, we performed oversampling on patches containing a single tissue type; solely normal, GP3, or GP4. The target count for each of these classes, *N*_*target*_, was set to 30% of the number of patches containing mixed pixel types, *N*_*mixedPixels*_. This ratio (0.3) was chosen to increase the representation of minority classes while mitigating the risk of model overfitting.

The number of synthetic patches to generate for each class, *N*_*add*_, was calculated by first defining the target count in Eq (1). Then, the number of patches to increase for each specific tissue type shown in Eq (2), is the difference between the target count and the original count, *N*_*tissueType*_ where $N_{expansion}\geq 0$. These additional *N*_*expansion*_ patches were generated by randomly duplicating existing patches (oversampling) from the corresponding class.

$$N_{target}=N_{mixedPixels}\times ratio$$ (1)

$$N_{expansion}=N_{target}-N_{tissueType}$$ (2)

**Fig 2:**
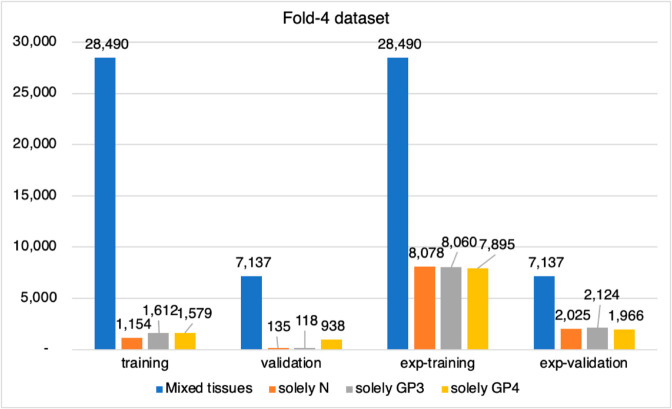
Distribution of patch pairs in Fold-4 dataset for training and validation sets, illustrating the imbalances and subsequent adjustments made through patch expansion.

In the subsequent step, we conducted a detailed pixel count and analysis for each slide, categorizing pixels into background, foreground, normal, GP3, and GP4. For example, Fig 2 illustrates the imbalanced proportions of patches found in both the training and validation sets of Fold-4 dataset. These compositions include: 1) mixed pixels of background, foreground, normal, GP3, and GP4; 2) solely normal pixels; 3) solely GP3 pixels; and 4) solely GP4 pixels.

**Fig 3:**
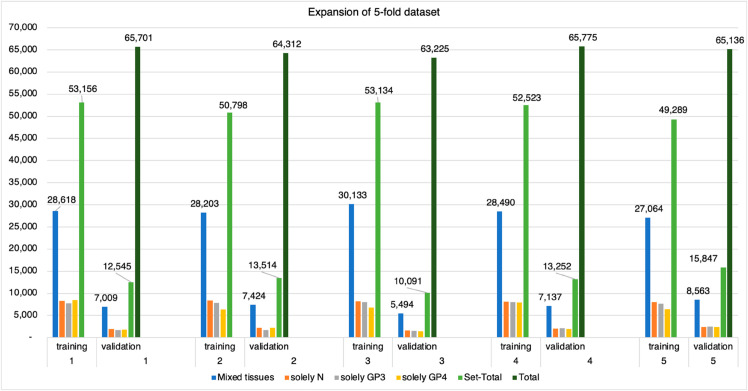
Quantitative expansion of the five-fold dataset, illustrating the total counts of patch pairs post-expansion for each fold in the training and validation sets, demonstrating the balancing process applied to the data.

As a result, the total count of patch pairs for each training and validation set were increased from 116,172 patches to approximately 160,000 patches, creating a more balanced dataset for model training as presented in Fig 3.

We applied the same preprocessing technique to the testing set. Importantly, we selected an optimal ratio of 0.20 to prevent overfitting, applying it exclusively to the unseen test set. This increased the number of patches from 12,218 to 15,638 (see S1 Fig. in the Supplementary Material).

The final step of data preprocessing involves a multi-class configuration. All pixels within the mask patches were classified into three distinct categories, as detailed in Table 1. In the implementation phase, the ground truth mask for each image patch was stored as a one-hot array. Class weights were calculated for all folds and applied to Dice loss, Jaccard coefficient, and Dice coefficient during the training phase (see Eqs 8 to 14). Additionally, dynamic data augmentation, including horizontal and vertical flips, both with selection probabilities of 0.5, was incorporated into the training process.

**Table 1:** Three class configuration for multi-class semantic segmentation.

Class	Description
0	Mixed pixels of background, foreground, and normal/benign
1	Pixels containing GP3 tissue
2	Pixels containing GP4 tissue

**Fig 4:**
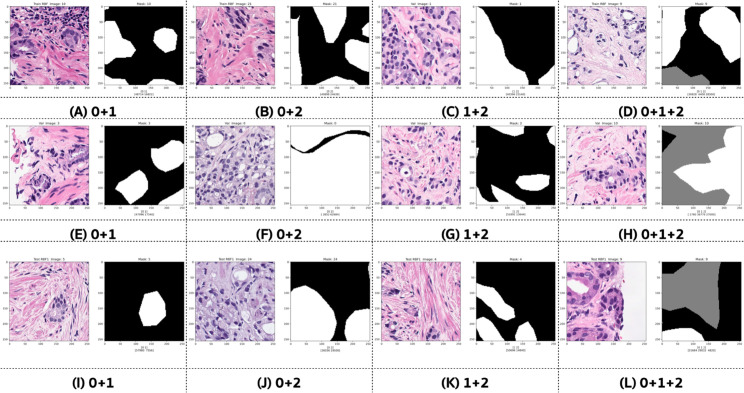
Examples of various mixed tissue patch pairs with their ground truth masks preprocessed into a multi-class configuration from Fold-4. Displayed are (A) - (D) for the training set, (E) - (H) for the validation set, and (I) - (L) for the test set.

Fig 4 illustrates examples of various mixed tissues in patch pairs. These images show ground truth masks preprocessed into a multi-class configuration from Fold-4. Panels (A) through (D) represent samples from the training set; (E) to (H) are from the validation set; and (I) to (L) are from the test set. In (A), (E), and (I), each image patch contains binary values representing the absence (zero) and presence (one) of GP3 tissues. In (B), (F), and (J), each image patch contains binary values representing the absence (zero) and presence (two) of GP4 tissues. In (C), (G), and (K), each image patch contains values indicating the presence of both GP3 and GP4 tissues. In (D), (H), and (L), each image patch contains values indicating the presence of normal, GP3, and GP4 tissues.

### Proposed method and model architecture

**Fig 5:**
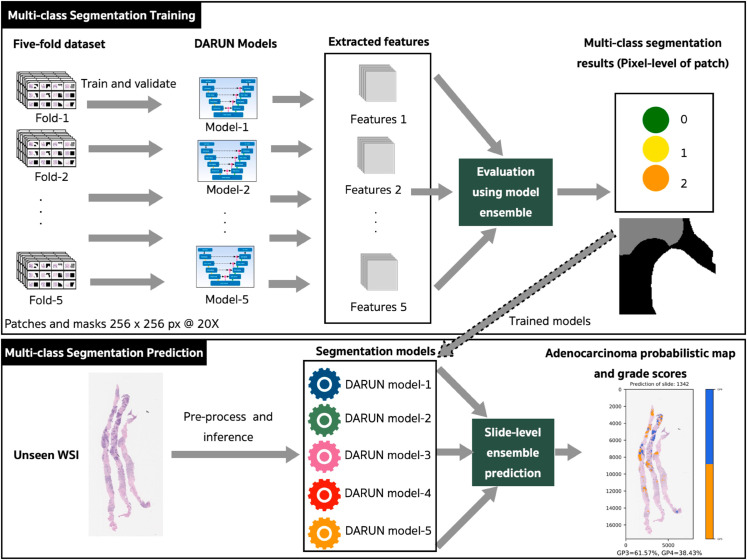
Proposed experimental design of multi-class semantic segmentation for prostate adenocarcinoma exploiting dilated attention-based residual convolutional U-Net (DARUN) models.

The proposed approach involves utilizing dilated attention-based residual convolutional U-Net (DARUN) models for multi-class semantic segmentation of benign, GP3, and GP4 prostate adenocarcinoma, as illustrated in Fig 5. During the training phase, the five-fold dataset—consisting of both training and unique validation sets for each fold—is input into the model for patch-level training and feature extraction. Subsequently, both the standard method (employing its respective trained model) and the model ensemble approach are applied to evaluate the validation set. This process culminates in pixel-level multi-class segmentation of each patch, encompassing classes zero, one, and two (see Table 1).

The architectural design of the attention-based residual convolutional blocks (RCBs) within the U-Net framework is depicted in S2 Fig. of the Supplementary Material. This model architecture takes advantage of the combination of attention gate units with RCBs [19], which incorporate DRs to enhance receptive fields [16]. These DRs are applied across all 2D layers of RCBs, situated in both the encoder and decoder paths.

Evaluation methods and trained models were thoroughly assessed to identify the most effective inference approach for the prediction stage. Statistical tests, as detailed in the Performance Analysis Approach section, were utilized to compare the performance of both the standard evaluation and the model ensemble evaluation.

In the prediction stage, 20 completely unseen WSIs from the prostate biopsy test set were used for adenocarcinoma segmentation. This process was employed to create slide-level prediction maps and grade the tissue scores accordingly.

### Configurations of training and validation

Real-time preprocessing was applied to image and mask patch pairs using a data generator before feeding them into the model for training.

The image patches were read using TensorFlow [20] and preprocessed using the SEResNet50 model [17,21]. This involved converting the color space from RGB to BGR and applying zero-centering with respect to the ImageNet [22] database for each color channel.

However, no rescaling of pixel values was performed for these image patches. The ground truth masks were converted on-the-fly into one-hot array labels to support the multi-class segmentation task.

The CNN-based layers in the model utilized kernel sizes of 3 x 3 pixels and employed the rectified linear unit (ReLU) activation function, as defined in Eq (3) [23]. The Softmax function as defined in Eq (4), was exclusively utilized in the prediction layer.

To address the unbalanced classes within each fold of the dataset, both Dice loss and categorical focal loss were utilized, as defined in Eq (8) and Eq (9), respectively. Additionally, the learning rate was configured to reduce based on the validation evaluation of the *Total*_*loss*_, as defined in Eq (10) for every epoch. The Adam optimizer, initialized with an optimal learning rate [21], was employed to minimize the total losses throughout the training epochs.

This training and validation process utilized fine-tuned hyperparameters, obtained from an exhaustive pilot study as reported in S1 Table of the Supplementary Material.

$$g_{(}\vec{z})=max(0,z)$$ (3)

$$\sigma (\vec{z}{)}_{j}=\frac{e^{z_{j}}}{\sum_{k=1}^{K}e^{z_{k}}}$$ (4)

### Performance evaluation metrics

Model performance for multi-class segmentation was evaluated at the pixel level using a standard set of metrics: Precision, Recall (Sensitivity), Accuracy, Jaccard Coefficient ($Jaccard^{\text{coef}}$), and Dice Coefficient ($Dice^{\text{coef}}$), as defined in Eq (5) through Eq (15). Our analysis was conducted across the five-fold cross-validation dataset (${𝒟}_{\text{val}}$) and the unseen test set (${𝒟}_{\text{test}}$).

We compared three distinct evaluation schemes:

- **Standard Evaluation:** Each validation fold is assessed only by its corresponding trained model (e.g., Fold-1 is predicted by Model-1).
- **Model Ensemble (ME) Evaluation:** Each of the five trained models generates a pixel-wise probability map for a given input. These five maps are then averaged to produce a final ensemble probability map, as shown in Eq (16). The predicted class for each pixel is then determined by applying an argument max operation (see Eq (17)).
- **Weighted Model Ensemble (WME) Evaluation:** This method extends the ME approach by multiplying the probability map from each model by a predetermined weight before averaging. The final prediction is similarly determined by an argument max operation on the resulting weighted probability map [17].

$$Precision=\frac{TP}{TP+FP}$$ (5)

$$Recall=\frac{TP}{TP+FN}$$ (6)

$$Accuracy=\frac{TP+TN}{TP+TN+FP+FN}$$ (7)

$$Dice_{loss}(Precision,Recall)=1-(1+\beta^{2})\frac{Precision\cdot Recall}{\beta^{2}\cdot Precision+Recall}$$ (8)

$$CategoricalFocal_{loss}(y_{true},y_{pred})=-y_{true}\cdot \alpha \cdot (1-y_{pred}{)}^{\gamma }\cdot \log(y_{pred})$$ (9)

$$Total_{loss}=Dice_{loss}+(1\cdot CategoricalFocal_{loss})$$ (10)

$$intersection(y_{true},y_{pred})=\sum_{i}\left|y_{true_{i}}\cdot y_{pred_{i}}\right|$$ (11)

$$union(y_{true},y_{pred})=\sum_{i}y_{true_{i}}+\sum_{i}y_{pred_{i}}-intersection$$ (12)

$$Jaccard^{\text{coef}}=\frac{intersection+smooth}{union+smooth}$$ (13)

$$Dice^{\text{coef}}=\frac{2\cdot intersection+\text{smooth}}{\sum_{i}y_{true_{i}}+\sum_{i}y_{pred_{i}}+\text{smooth}}$$ (14)

$$𝒫=\{{\text{Precision}}_{j},{\text{Recall}}_{j},{\text{Accuracy}}_{j},Jaccard_{j}^{\text{coef}},Dice_{j}^{\text{coef}}\},j\in \{0,1,2\}$$ (15)

$${\bar{p}}_{i,j}({𝒟}_{\text{set}})=\frac{1}{F}\sum_{f=1}^{F}p_{i,j}^{f}({𝒟}_{\text{set}}),\;\text{set}\in \{\text{val},\text{test}\},\;j\in \{0,1,2\}$$ (16)

$${\hat{y}}_{i}=\arg\max_{j}({\bar{p}}_{i,j}),\;j\in \{0,1,2\}$$ (17)

### Performance analysis approach

To determine if observed performance differences were statistically significant, we employed the non-parametric Wilcoxon signed-rank test [24,25]. This method is more robust than a paired t-test for our analysis, given the small sample size (N = 5 folds) and the lack of assumption about normal data distribution.

We applied a one-sided version of the test to three paired-group scenarios, calculating the difference, *D*_*f*_, between the performance metrics (*P*) for each fold (*f*):

1. **Standard vs. ME**:
  $$D_{1,f}=P_{\text{ME},f}-P_{\text{Std},f}$$ (18)
2. **ME vs. WME**:
  $$D_{2,f}=P_{\text{WME},f}-P_{\text{ME},f}$$ (19)
3. **DR-enabled vs. DR-disabled**:
  $$D_{3,f}=P_{\text{DR-enabled},f}-P_{\text{DR-disabled},f}$$ (20)

The test operates on these differences by ranking their absolute values and then summing the ranks for positive (*W*^ + ^) and negative (*W*^−^) differences separately. The resulting test statistic, *W*, is the minimum of these two sums:

$$W=\min(W^{+},W^{-})$$ (21)

This *W* statistic and its corresponding p-value were used to quantify the statistical significance of the performance difference between the paired groups.

### Validation and robustness experimental designs

To assess the model’s real-world applicability, we designed two additional experiments. First, an external validation was planned to evaluate the model’s generalization capabilities on data from a different source. For this, we selected the Cancer Moonshot Biobank - Prostate Cancer Collection (CMB-PCA) [26]. Second, to test the architectural robustness of our proposed model, we planned to evaluate its performance on an unrelated medical imaging task using a subset of the Skin Cancer HAM10000 dataset [27].

## Results

This section presents our experimental findings, beginning with a quantitative analysis to determine the most effective evaluation strategy. We first compare the performance of the standard, ME, and WME evaluation methods. We then apply the best-performing method to generate and assess slide-level semantic segmentation maps for GPs 3 and 4 on both validation and unseen test data. The section concludes with an external validation and an assessment of the model’s robustness.

### Model ensemble performance analysis

We compared the performance of a standard evaluation against the ME approach across the five validation folds. In the standard method, each fold was assessed only by its corresponding trained model (e.g., Fold-1 by Model-1). In contrast, the ME approach assessed each fold using an ensemble of all five trained models.

As detailed in Table 2, the ME method demonstrated consistently superior performance over the standard method. To quantify this improvement, a Wilcoxon signed-rank test was performed on the paired mean scores. The test confirmed that the ME evaluation method was significantly better than the standard method (W = 0, $p=3\times 10^{-8}$).

**Table 2:** Comparison of mean performance metrics between the standard and ME evaluation methods across the five cross-validation folds.

Metric	Fold	Standard	ME
Jaccard	1	0.647	0.762
	2	0.565	0.765
	3	0.556	0.770
	4	0.516	0.766
	5	0.585	0.770
Dice	1	0.783	0.864
	2	0.718	0.867
	3	0.707	0.869
	4	0.678	0.867
	5	0.734	0.870
Precision	1	0.794	0.865
	2	0.734	0.870
	3	0.703	0.874
	4	0.686	0.887
	5	0.740	0.877
Recall	1	0.777	0.865
	2	0.709	0.864
	3	0.712	0.866
	4	0.673	0.853
	5	0.732	0.864
Accuracy	1	0.870	0.914
	2	0.825	0.913
	3	0.827	0.920
	4	0.800	0.915
	5	0.840	0.916

The ME method shows a clear and consistent improvement over the standard method across all metrics. A Wilcoxon signed-rank test confirms this difference is statistically significant (W = 0, $p=3\times 10^{-8}$).

**Fig 6:**
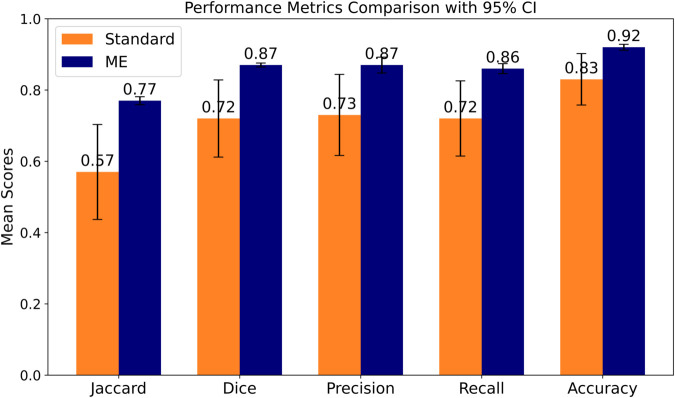
Comparison of mean performance metrics between the standard and ME evaluation methods across the five validation folds. Error bars represent 95% confidence intervals. The ME method demonstrates a clear and statistically significant improvement over the standard method across all metrics (Wilcoxon signed-rank test, W = 0, $p=3\times 10^{-8}$).

Fig 6 presents average performance scores with 95% of confident intervals (CI) for each metric across the five-fold validation sets, comparing the standard evaluation method and the ME method. The ME performances showcased better average metric values compared to those of the standard evaluations. The overall accuracy reached 0.92, representing the highest performance across the validation sets.

On the unseen test set of 20 WSIs, the ME method demonstrated robust overall classification performance, achieving a mean accuracy of 0.811 with a narrow 95% confidence interval of [0.743 - 0.880]. This indicates a reliable ability to correctly classify the majority of tissue pixels. However, when evaluating the precision of the segmentation boundaries using the Dice coefficient, the performance was more modest at a mean of 0.637. The corresponding wide 95% confidence interval of [0.164 - 1.111] reveals significant performance variability across the different Gleason patterns.

The model particularly struggled to accurately delineate the boundaries of GP3, given its morphological similarity to benign tissue. This highlights a key limitation and an important direction for future work aimed at improving segmentation consistency. The complete performance metrics and their corresponding 95% CI are detailed in Table 3.

**Table 3:** Mean performance of the ME method on the unseen test set (N=20 WSIs).

Metric	Mean	95% CI
Jaccard	0.487	[0.000 - 0.997]
Dice	0.637	[0.164 - 1.111]
Precision	0.692	[0.578 - 0.806]
Recall	0.627	[0.000 - 1.255]
Accuracy	0.811	[0.743 - 0.880]

The mean and 95% CI were calculated based on the performance across the three output classes (benign, GP3, and GP4).

### Weighted model ensemble performance analysis

To investigate if performance could be further improved, we evaluated the WME against the standard ME. We performed an exhaustive grid search to find the optimal weights for the five trained models, using a 50% subset of the Fold-3 validation set due to computational constraints. The optimal weights were found to be 0.9, 0.5, 0.5, 0.5, and 0.5 for the DARUN-1 through DARUN-5 models, respectively.

As shown in Table 4, the WME method yielded a consistent and statistically significant improvement over the standard ME on this validation subset (W = 0, $p=3.1\;\times \;10^{-5}$). However, when this optimized WME was applied to a subset of the unseen testing data (approx. 5,000 patches), its performance was inconsistent compared to the standard ME. Given that the WME did not generalize as robustly to completely new data, we selected the simpler, more stable ME method for all final inferences on the testing set.

**Table 4:** Class-wise performance comparison between the ME and WME methods on the Fold-3 validation set.

Metric	Class	ME	WME
Jaccard	0	0.834	0.853
	1	0.735	0.762
	2	0.742	0.762
Dice	0	0.909	0.920
	1	0.847	0.865
	2	0.852	0.865
Precision	0	0.899	0.909
	1	0.880	0.891
	2	0.842	0.865
Recall	0	0.920	0.932
	1	0.816	0.841
	2	0.861	0.865
Accuracy	0	0.906	0.917
	1	0.931	0.939
	2	0.925	0.932

The WME method demonstrates a statistically significant improvement over the ME method (W = 0, $p=3.1\;\times \;10^{-5}$).

### Segmentation results and grading score

**Fig 7:**
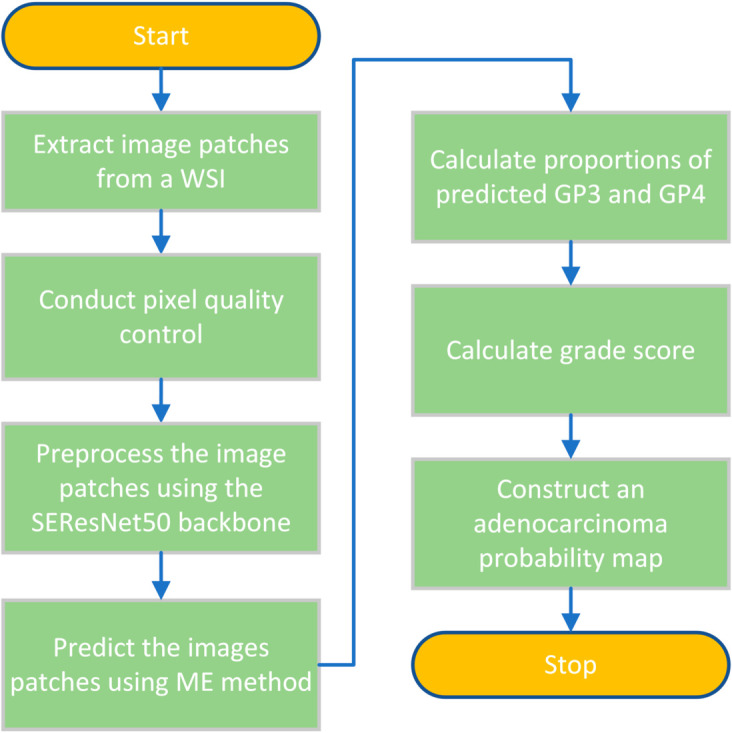
Inference steps outlining the process for generating adenocarcinoma probability maps specifically targeting GP3 and GP4 tissues.

In this section, we applied the ME method to compute GS and generate adenocarcinoma probability maps, distinguishing between GP3 and GP4. The inference process followed the steps outlined in Fig 7.

A WSI is divided into image patches sized 256 x 256 in RGB color mode. Patches with a low pixel ratio (<50%) are filtered out to maintain pixel quality. These filtered patches undergo preprocessing using the SEResNet50 backbone. Subsequently, the image patches are predicted and the classes are evaluated using the ME method. Predicted GP3 and GP4 pixels, representing dominant regions of both patterns, are utilized to calculate the proportions of the patterns and determine the GS. Finally, an adenocarcinoma probability map for GP3 and GP4 is constructed for the WSI.

#### Segmentation for the validation set.

Slide ID 1925 from the Fold-4 validation set was selected for inference, with the resulting segmentation shown in S3 Fig. of the Supplementary Material. This slide contained approximately eight biopsy cores, covering all classes such as benign, GP3, and GP4 tissues (A). Using the standard prediction method (B), GP4 was the dominant classification, comprising 99.90% of the segmented areas. In contrast, the score proportions for GP3 and GP4 were more balanced and reflective of the ground truth, with a ratio of 31:69 (C), respectively.

The ME method demonstrated robust performance in identifying benign or negative areas. Nonetheless, there were false predictions noted for GP3, indicated by green and orange rectangles in the figure. The detection of GP3 proved to be more challenging due to its characteristically poorly-formed glands and a reduced presence of well-formed glandular structures.

#### Segmentation for the testing set.

**Fig 8:**
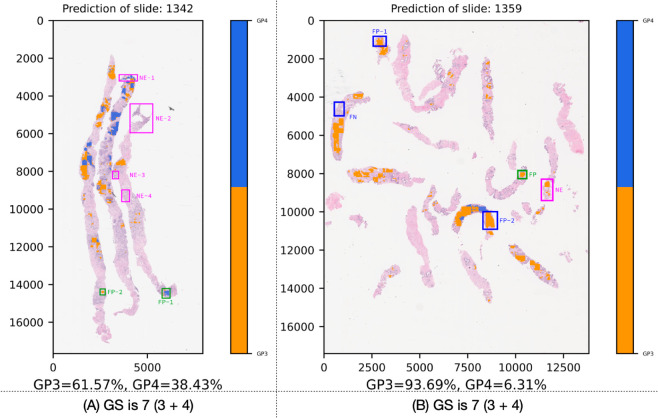
Adenocarcinoma semantic segmented results for slide ID 1342 and 1359; GS for Gleason scores, NE for not evaluated, FP for false positive, and FN for false negative.

An unseen slide, ID 1342 from the testing set, was randomly selected for inference to create the adenocarcinoma semantic segmentation map and calculate the GS, as presented in Fig 8(A). The differentiation of adenocarcinoma between GP3 and GP4 indicated approximately 62% for GP3 and 38% for GP4. Upon thorough inspection, the region marked NE-1 was not evaluated (NE) by our first pathologist due to doubts, requiring a consultation with our second pathologist. Using the ME method, the models segmented the region into GP3 and GP4, which appeared reasonable. However, the subsequent regions marked NE-2, NE-3, and NE-4 exhibited fluctuating predictions, suggesting benign tissue. Meanwhile, FP-1 indicates a false positive for benign tissue, but the models predicted it as predominantly GP4 with a small presence of GP3. Similarly, FP-2, while showing a false positive for benign tissue, actually corresponds to GP3.

Fig 8(B) showcases the adenocarcinoma semantic segmentation map and GS of an unseen slide, ID 1359, from the testing set. The GS revealed approximately 94% for GP3 and 6% for GP4. Upon careful inspection, FP-1 indicates a falsely positive segmented area as GP4, which was actually a mixture of poorly-formed glands from both GP3 and GP4. Additionally, FP-2 indicates another false positive prediction, mistakenly identified as GP4 in a different area. The blue FN marker indicates tissue that was falsely segmented as GP4, whereas it was actually benign. Additionally, the green FP marks an area where benign tissue was incorrectly identified as GP3 by the models. The final notable result, marked in pink, corresponds to the ground truth label of NE. In our preprocessing steps, tissues annotated as NE were excluded from this study. These tissues were deemed uncertain by the pathologists, as their pathological characteristics were not distinct or refined enough for a confident label.

Additionally, the models incorrectly classified the tissue within the pink frame as GP3, even though it resembled benign tissue. This misclassification led to an inflated GP3 score. Nevertheless, our pathologists considered the slide-level segmentation and GS acceptable.

### External validation

To evaluate the model’s generalization capabilities, we applied our trained ensemble to the external CMB-PCA dataset [26]. The ME method was used to segment the slides and calculate the Gleason Score, allowing us to assess performance stability on data from a different source.

For this analysis, we selected a subset of the CMB-PCA dataset, comprising 10 slides from eight patients. We specifically chose four slides that contained only adenocarcinoma tissues for examination. The ME method of the models was applied to segment these slides and calculate the GS. The results of this analysis are summarized in Table 5.

**Table 5:** Summary of Gleason pattern percentages and detected grade scores for prostatic adenocarcinoma slides in the CMB-PCA Dataset.

Slide ID	Percent tumor*	GP percentages**	Grade score
MSB-02917-01-02	50% - 69%	GP3 = 6%, GP4 = 94%	7 (4 + 3)
MSB-03973-01-02	20% - 49%	GP3 = 17%, GP4 = 83%	7 (4 + 3)
MSB-05563-01-02	20% - 49%	No GP3 or GP4 tissue was detected.	N/A
MSB-08178-01-02	50% - 69%	GP3 = 22%, GP4 = 78%	7 (4 + 3)

* The percentages were evaluated from all biopsy cores in the slide.

** The Gleason pattern percentages were evaluated from all regions which were predicted into GP3 and GP4.

**Fig 9:**
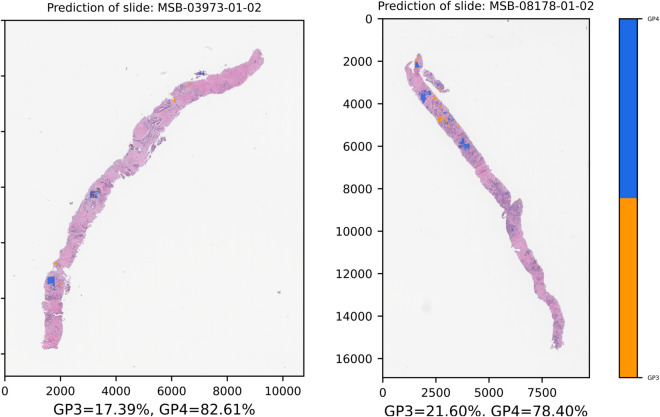
Examples of successful adenocarcinoma segmentation on the external CMB-PCA validation dataset. The figure displays the results for slide ID MSB-03973-01-02 and MSB-08178-01-02, both of which were assigned a Gleason Score of 7 (4+3).

Fig 9 displays examples of successful segmentation on the external dataset. For slide ID MSB-03973-01-02, the model predicted a GP4-dominant GS of 7 (4+3), which aligns with the ground truth range of 20-49% tumor nuclei. Similarly, for slide ID MSB-08178-01-02, the model also assigned a GS of 7 (4+3), corresponding to the ground truth of 50-69% tumor nuclei. The segmentation results for a third successfully identified slide, MSB-02917-01-02, are presented in S4 Fig. (A) of the Supplementary Material. It is important to note, however, that the model produced one false negative. For slide ID MSB-05563-01-02, no GP3 or GP4 tissue was detected, despite the ground truth indicating 20-49% adenocarcinoma as presented in S4 Fig. (B) of the Supplementary Material.

Notably, we cannot directly compare these numbers due to their different assessment methods. Our proposed GS calculation was based solely on all predicted regions of both patterns, which might result in false negative predictions when compared to an external validation source. If we were to compare them qualitatively, the graded regions in our segmentation might appear more extensive than they are visually. Nonetheless, most of these slides were predicted as true positives at the slide level or patient level, which lends credibility and acceptability to our results.

### Model robustness

To test the architectural robustness of our model, we evaluated its performance on an unrelated task using a subset of the Skin Cancer HAM10000 dataset [27]. This experiment demonstrates the model’s ability to perform well on different types of medical images. The code and trained model for this task have been made publicly available as a Kaggle notebook to ensure reproducibility [28].

Performance evaluations of the model achieved an average Jaccard coefficient of 0.94 and a Dice coefficient of 0.97 for the validation set, and 0.91 and 0.95 for the test set, respectively. Based on these results, we can confidently assert that the proposed architecture demonstrates acceptable model robustness.

## Discussion

This section discusses the experimental findings, including the ablation study of dilation rate configuration, comparison to relevant works, computational performance and clinical workflow integration, and limitations.

### Ablation study of dilation rate configuration

**Fig 10:**
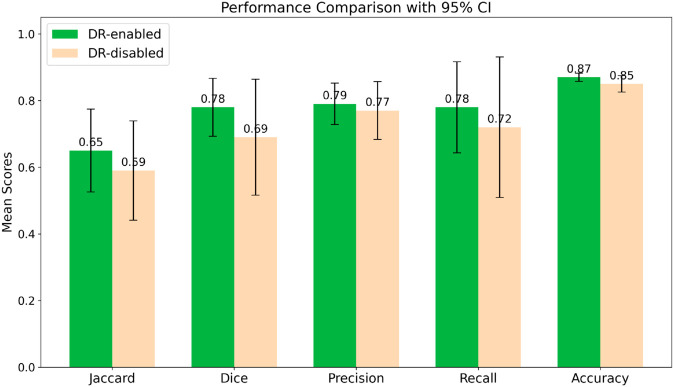
Comparison of performance metrics: DR-enabled vs. DR-disabled models for the Fold-1 dataset. The DR-enabled model performed significantly better across all metrics, with a p-value of 0.0062 (*W* = 17.5).

We conducted an ablation study to evaluate the impact of using DRs by comparing the performance of our model with and without this feature on the Fold-1 validation set. Fig 10 displays the mean performance metrics with their corresponding 95% confidence intervals. To assess if the observed improvements were statistically significant, we performed a one-sided Wilcoxon signed-rank test on the 15 paired performance metrics across all classes.

The test revealed a statistically significant difference between the two configurations (W=17.5,p=0.0062). This result confirms that the DR-enabled model significantly outperforms the DR-disabled model. Therefore, our experiments demonstrate that using dilated convolutions is essential for expanding the receptive fields of the feature maps, leading to improved performance in this segmentation task.

### Comparison to relevant works

We are the first team in Thailand to analyze our own collected digitized prostate WSIs using AI for semantic segmentation of PCa. However, we indirectly compared our results to related works, especially those focused on the semantic segmentation of PCa, as presented in Table 6. We selected the related works based on similar dataset characteristics, such as H&E staining and 20X magnification. Our proposed method with the ME evaluation in this study performed better than the related works in terms of the Dice coefficient, precision, and overall accuracy.

**Table 6:** Comparison of model performance metrics with those of related works.

Study	Dice	Jaccard	Precision	Recall	Accuracy
Li et al. [13]	-	0.77	-	-	0.90
Marginean et al. [14]	-	-	-	0.89 for GP3; 0.91 for GP4	-
Ahmad et al. [15]	0.84	0.78	0.86	0.84	-
Proposed method	0.87	0.77	0.87	0.86	0.92

### Limitations

Primarily, the experiments were conducted using a dataset of 100 WSIs originating from a single institution and scanner. This homogeneous data source inherently limits the generalizability of our findings and likely contributed to challenges like overfitting, despite mitigation efforts such as organizing training and validation slides into five-fold cross-validation sets, encompassing approximately 160,000 patches (including images and masks). While our method achieved optimal and satisfactory results within this dataset, the constrained data variability represents a significant limitation.

To address these concerns, future work should prioritize expanding the dataset to include a larger volume of data and more diverse cohorts, specifically from multi-institutional sources and different scanner types. Strategies such as stain normalization [29] or advanced domain adaptation techniques, like self-supervised learning (SSL) [30], could also be investigated to mitigate scanner bias and enhance model robustness. Ultimately, validating the model across multi-center datasets is crucial to ensure consistent behavior and clinical reliability. Similarly, our external validation on the CMB-PCA dataset was qualitative, as pixel-level annotations were not available to compute quantitative metrics, highlighting the need for publicly available test sets with detailed ground truth masks.

The second limitation involves the annotations. These were initially performed by a single pathologist; however, when uncertainty arose, a second expert opinion was sought to reach a consensus. It is important to note that while this pathologist has over 30 years of experience in the field, no formal assessment of inter-observer variability or inter-observer agreement metrics (e.g., Cohen’s kappa) was recorded during the annotation process.

The third limitation concerns the performance of our models in the multi-class segmentation task. We assessed the performance of each class independently, which sometimes led to uncertainties, particularly in differentiating between benign and GP3 tissues due to their similar prostate gland formations. To address this specific challenge of class ambiguity, we have recently proposed a framework using adaptive thresholding to optimize predictions for clinical deployment in the PCa domain [31]. Despite this challenge, the overall model performance showed promising trends, laying a foundation for future enhancements.

Fourth, our multi-class segmentation task primarily focused on differentiating adenocarcinoma between GP3 and GP4 due to the prevalence of these patterns in our biopsy data. While our method effectively calculates GS for adenocarcinoma, the presence of GP5 tissues can lead to false detection in biopsy cores or region segmentation. Therefore, extending this segmentation task to include GP5 tissues remains a challenge for our future research. Another learning technique from similar pathological datasets such as SSL has caught our interest [30,32]. In addition, promising models such as the ViT [8,33], UNet-like pure transformer [34], transformer models with shifted windows mechanisms [35], are promising directions for future investigation.

Lastly, the methods proposed in this paper did not directly address grading related to clinical outcomes or radical prostatectomy for patients. Predicting a slide’s characteristics is just one factor in suggesting treatment or remedies for a patient.

### Computational performance and clinical workflow integration

The integration of this AI tool into a pathology workflow must be feasible. This depends on both the computational requirements and the time needed to analyze a slide (inference time).

Training our five models was computationally intensive, requiring approximately 38 hours on a high-performance cluster equipped with an NVIDIA A100 GPU, or around six days on a local workstation with an NVIDIA RTX 5000 GPU. However, this training process is a one-time, offline task. The more critical factor for clinical adoption is the inference performance on unseen cases.

While not formally benchmarked, we can estimate the inference time. A typical WSI in our test set contains between 5,000 and 15,000 relevant tissue patches. On a modern clinical-grade workstation GPU (such as the NVIDIA RTX 5000), processing a single patch takes a fraction of a second. Therefore, we estimate the total time to analyze a full WSI, including patch extraction, preprocessing, prediction, and reconstruction of the segmentation map, to be between 10 to 20 minutes.

This turnaround time makes integration into a clinical workflow highly feasible. The model could run automatically overnight on newly scanned slides, providing pathologists with a pre-analyzed case. The generated segmentation map would serve as an efficient screening tool, immediately indicating regions of interest (GP3 and GP4), and providing an initial GS. Rather than replacing the pathologist, this tool serves as a powerful assistive tool, helping to focus their expertise and reduce inter-observer variability. Future work will involve deploying this model as a web application to enhance its accessibility in a clinical setting.

## Conclusion

In this work, we highlighted the critical importance of detection and grading of adenocarcinoma tissues in prostate glands, specifically distinguishing between Gleason patterns 3 and 4. This differentiation is crucial for enhancing survival rates and improving patients’ quality of life.

We proposed a multi-class pixel-level semantic segmentation method for detecting adenocarcinoma in PCa. A publicly available dataset of 100 digitized whole-slide images was introduced, divided into 64 for training, 16 for validation, and 20 for testing.

The dataset includes image patches and corresponding ground truth masks. To address class imbalance among benign, GP3, and GP4, we performed pixel expansion. A five-fold cross-validation method was implemented, organizing training and unique validation sets. Class weights were also computed and applied during training. We introduced the proposed model, an architecture that combines dilated attention with a residual convolutional U-Net. Hyperparameters were finely tuned to optimize performance.

The superiority of the chosen ME method was statistically validated using the non-parametric Wilcoxon signed-rank test. On the validation folds, the model achieved high performance with a mean Dice coefficient of 0.87 and an accuracy of 0.92. When applied to the unseen test set, the model yielded a robust mean accuracy of 0.81, though the mean Dice coefficient was more modest at 0.64, reflecting the challenges in maintaining precise segmentation consistency on new data. Furthermore, the model’s architecture demonstrated excellent robustness, achieving a Dice coefficient of 0.95 in tests on a separate, unrelated public dataset.

The ensemble method was selected for inferring unseen testing slides, constructing adenocarcinoma segmentation, and calculating GS for GP3 and GP4 at the slide level. The pathologists were satisfied with the results for qualitative measurements at the slide level. Based on our dataset and experimental results, we conclude that our method could serve as an optimal screening tool for PCa detection in current clinical settings.

## Supporting information

S1 Fig Patch pair counts in the test set before and after expansion. (TIF)

S2 Fig Enhanced model architectural design of the proposed DARUN model. (TIF)

S3 Fig Adenocarcinoma segmentation prediction and GP percentages for slide ID 1925 of Fold-4 validation set; black indicates TN for benign, orange indicates FN for GP3, and green indicates FP for benign. (TIF)

S4 Fig Segmentation results on the external CMB-PCA validation dataset. The figure shows (A) a successful prediction for slide ID MSB-02917-01-02 and (B) a false negative prediction for slide ID MSB-05563-01-02.
(TIF)

S1 Table Hyperparameters and their configurations for the proposed DARUN model. (DOCX)
