# Supplementary figures and images for: Detection and score grading for prostate adenocarcinoma using semantic segmentation
Source: PLoS One. 2025 Sep 19;20(9):e0331613. doi: 10.1371/journal.pone.0331613 (PMC12448973; doi:10.1371/journal.pone.0331613)

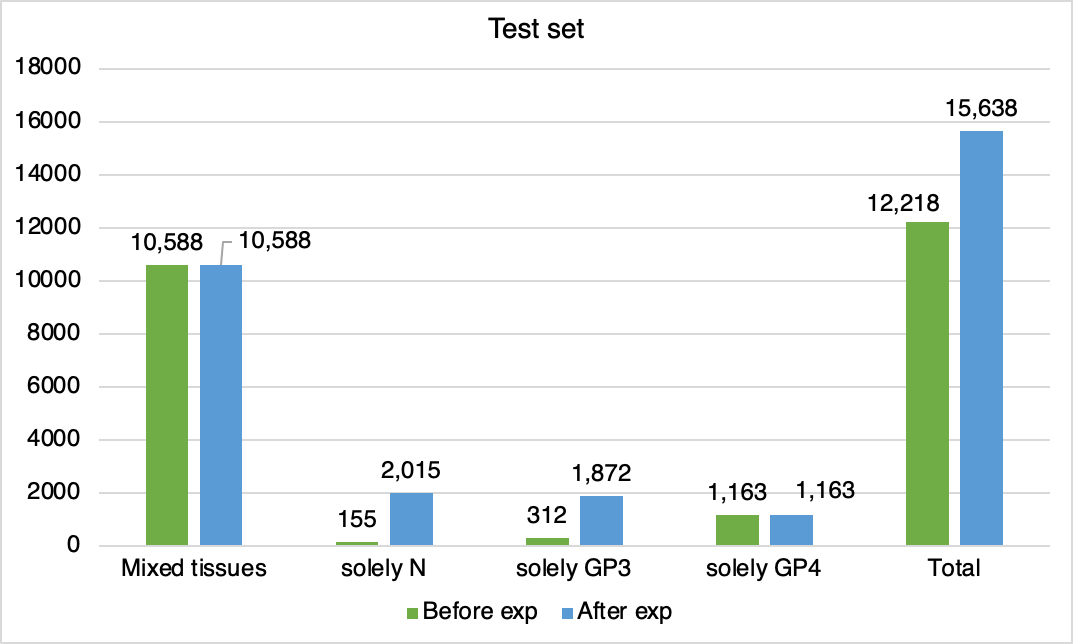

Supplement: S1 Fig — (TIF) [file pone.0331613.s001.tif]

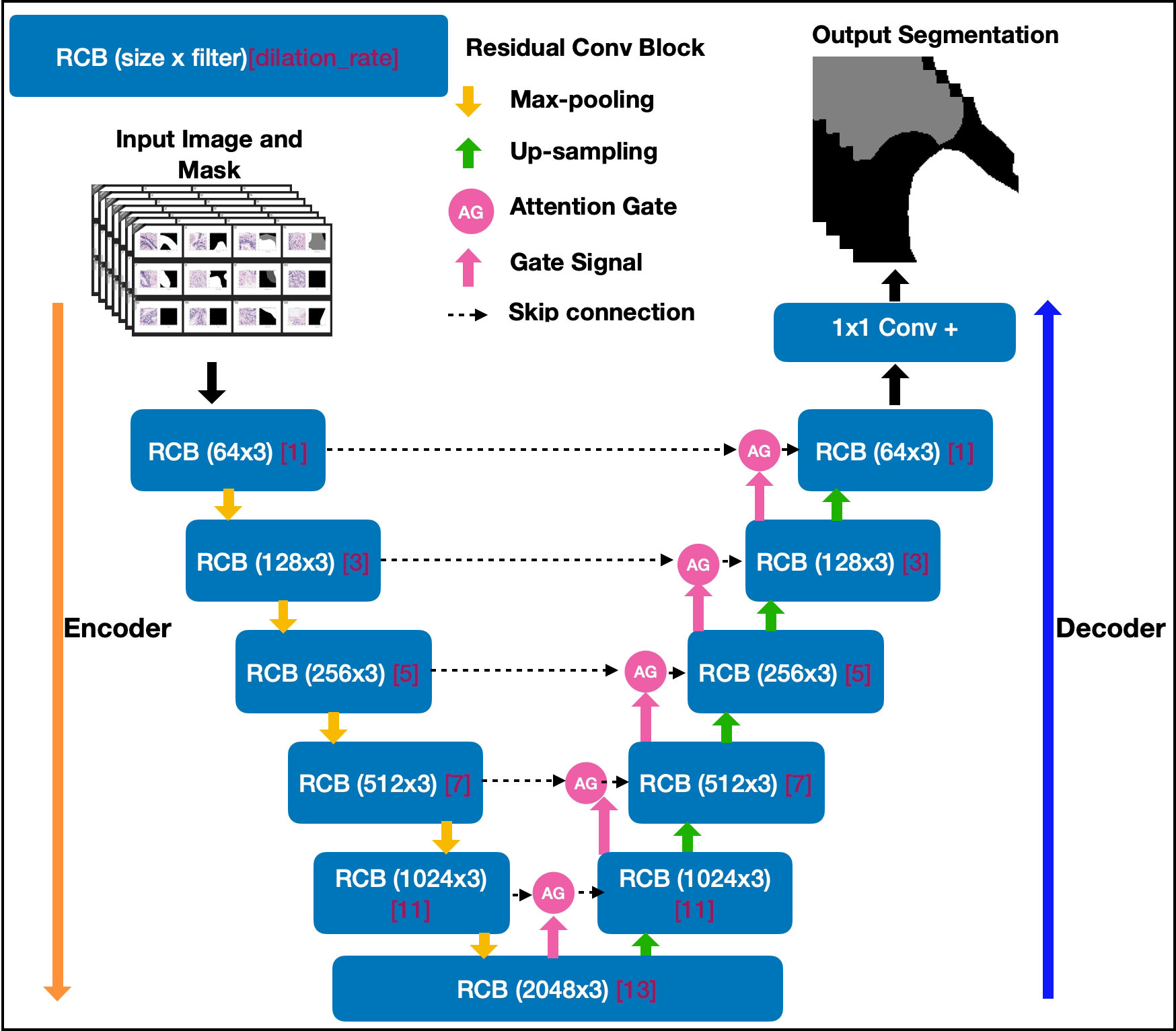

Supplement: S2 Fig — (TIF) [file pone.0331613.s002.tif]

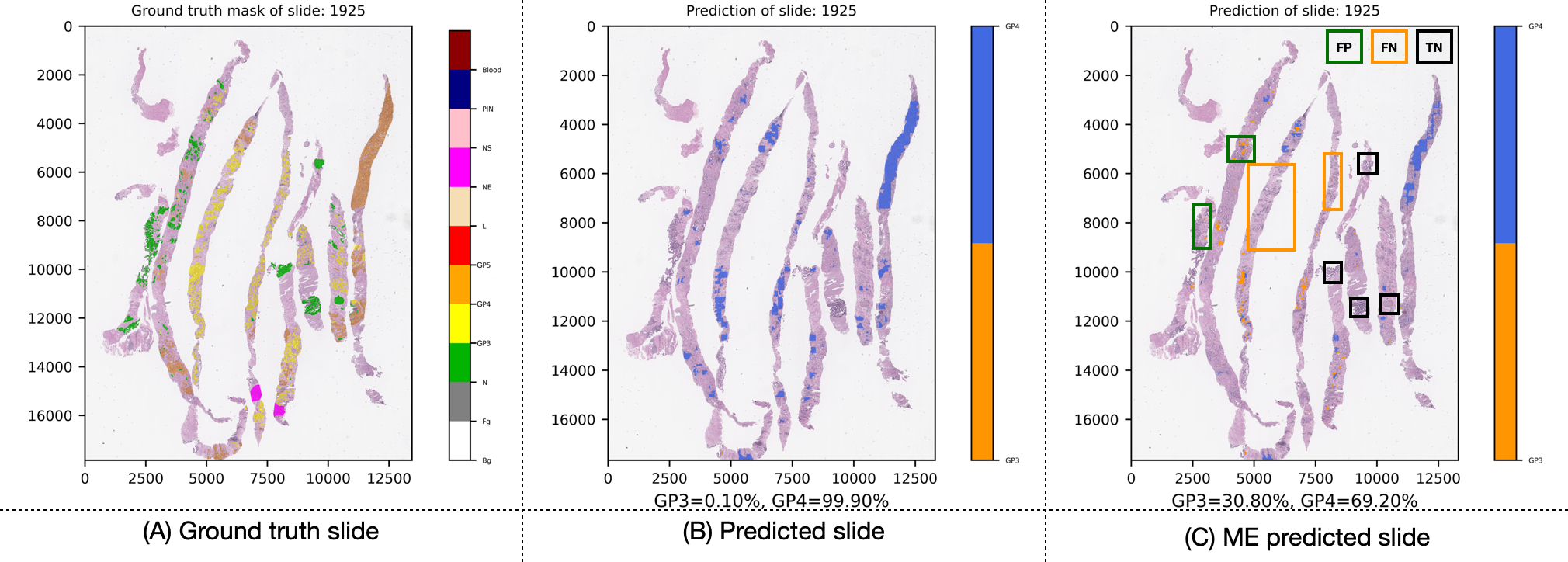

Supplement: S3 Fig — (TIF) [file pone.0331613.s003.tif]

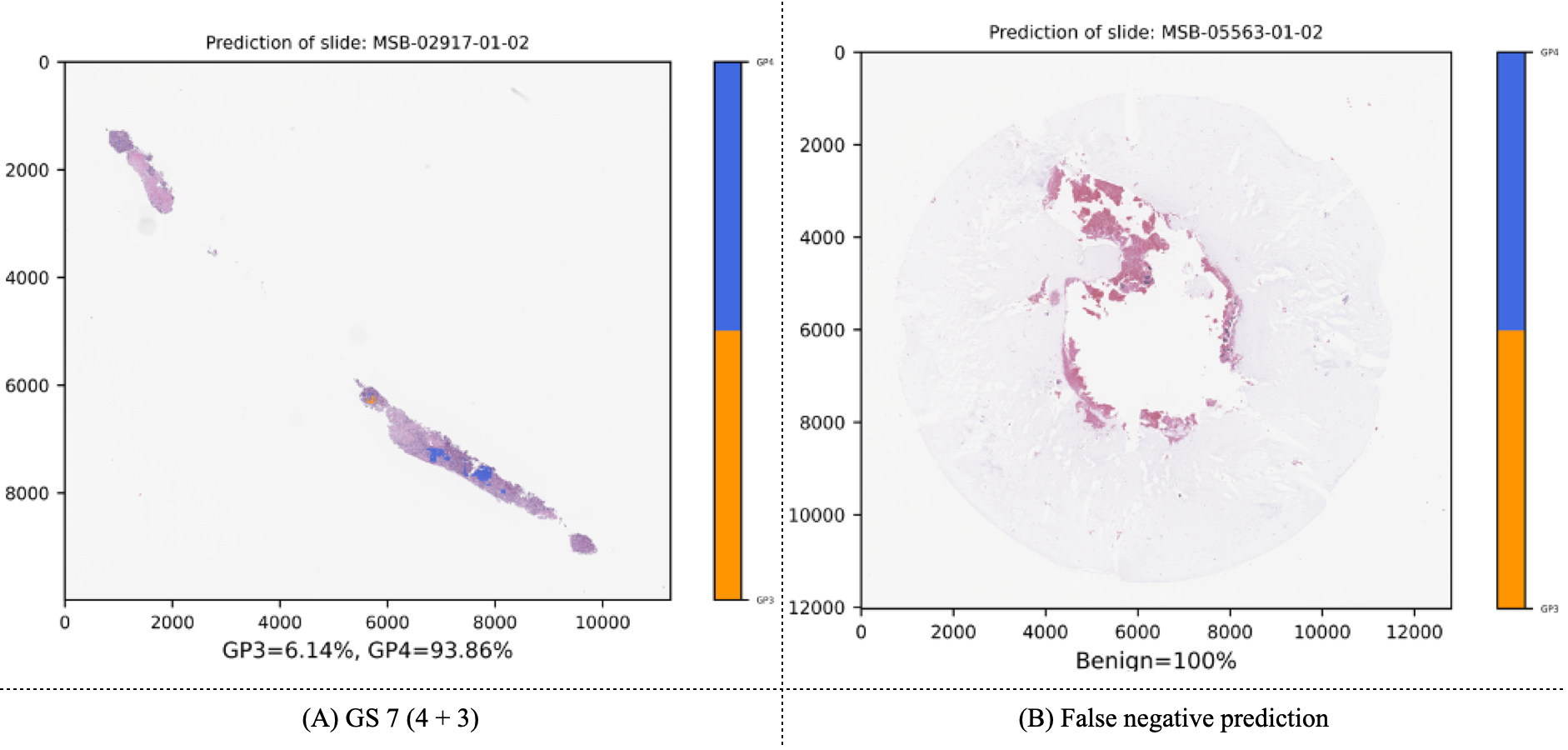

Supplement: S4 Fig — The figure shows (A) a successful prediction for slide ID MSB-02917-01-02 and (B) a false negative prediction for slide ID MSB-05563-01-02. (TIF) [file pone.0331613.s004.tif]
