# Supplementary material for: Detection and score grading for prostate adenocarcinoma using semantic segmentation
Source: PLoS One. 2025 Sep 19;20(9):e0331613. doi: 10.1371/journal.pone.0331613 (PMC12448973; doi:10.1371/journal.pone.0331613)
Supplement: S1 Table — (DOCX) [file pone.0331613.s005.docx]

**S1 Table. Hyperparameters and their configurations for the proposed DARUN model.**

| **Parameter** | **Configuration** |
| --- | --- |
| Image patch preprocessing | Utilized the SEResNet50 of Segmentation Models version 1.0.1 |
| Patch size | 256 x 256 |
| Kernel size | 3 x 3 |
| Dilation rate | 1, 3, 5, 7, 11, 13 |
| Batch size | 8 |
| Epochs | 120 |
| Dropout rate | 0.25 |
| Activation function | ReLu for all CNN-based layers and Softmax for the prediction layer |
| Loss function | Defined in the Equation 8 where alpha=0.25, beta=1.0, gamma=2.0 |
| Optimizer | Adam with initialized learning rate |
| Learning rate (LR) | 1e-4 |
| Class weights | Computed |
| Data augmentation | Horizontal flip (selection probability of 0.5) and vertical flip (selection probability of 0.5) |
| ReduceLROnPlateau | Monitor on validation of the Total loss with factor = 0.8, patience = 5, min_lr = LR/Epochs |
